# Supplementary material for: A Blue Light-Responsive Strong Synthetic Promoter Based on Rational Design in Chlamydomonas reinhardtii
Source: Int J Mol Sci. 2023 Sep 27;24(19):14596. doi: 10.3390/ijms241914596 (PMC10572394; doi:10.3390/ijms241914596)
Supplement: Supplementary file 1 [file ijms-24-14596-s001.zip › ijms-2610307-supplementary.pdf]

Supplementary Table S1. The sequences of *GUS, cadA*, and the native promoters used in the study.

Name	5’ - 3’ sequence
GUS **gene**: 1812bp	ATGCTGCGCCCCGTGGAGACCCCCACCCGCGAGATCAAGAAGCTGGACGGCCTGTGGGC<br>CTTCAGCCTGGACCGCGAGAACTGCGGCATCGACCAGCGCTGGTGGGAGAGCGCCCTGC<br>AAGAGAGCCGCGCGATCGCGGTGCCCGGCAGCTTCAACGACCAGTTCGCGGACGCCGA<br>CATCCGCAACTACGCGGGCAACGTGTGGTACCAGCGCGAGGTGTTCATCCCCAAGGGCT<br>GGGCGGGCCAGCGCATCGTGCTGCGCTTCGACGCGGTGACCCACTACGGCAAGGTGTGG<br>GTGAACAACCAGGAGGTCATGGAGCACCAGGGCGGCTACACCCCCTTCGAGGCGGACG<br>TGACCCCCTACGTGATCGCCGGCAAGAGCGTGCGCATCACCGTGTGCGTGAACAACGAG<br>CTGAACTGGCAGACCATCCCCCCCGGCATGGTCATTACCGACGAGAACGGCAAGAAGAA<br>GCAGAGCTACTTCCACGACTTCTTCAACTACGCCGGCATCCACCGCAGCGTGATGCTGTA<br>CACCACCCCCAACACCTGGGTGGACGACATCACCGTGGTGACCCACGTGGCCCAGGACT<br>GCAACCACGCGAGCGTGGACTGGCAGGTGGTGGCCAACGGCGACGTGAGCGTGGAGCT<br>GCGCGACGCCGACCAGCAGGTGGTGGCGACCGGCCAGGGCACCAGCGGCACCCTGCAA<br>GTGGTGAACCCCCACCTGTGGCAGCCCGGCGAGGGCTACCTGTACGAGCTGTGCGTGAC<br>CGCCAAGAGCCAGACCGAGTGCGACATCTACCCCCTGCGCGTGGGCATCCGCAGCGTGG<br>CCGTGAAGGGCGAGCAGTTCCTGATCAACCACAAGCCCTTCTACTTCACCGGCTTCGGC<br>CGCCACGAGGACGCCGACCTGCGCGGCAAGGGCTTCGACAACGTGCTGATGGTGCACG<br>ACCACGCGCTGATGGACTGGATCGGCGCCAACAGCTACCGCACCAGCCACTACCCCTAC<br>GCGGAGGAGATGCTGGACTGGGCGGACGAGCACGGCATCGTGGTCATTGACGAGACCG<br>CCGCGGTGGGCTTCAACCTGAGCCTGGGCATCGGCTTCGAGGCGGGCAACAAGCCCAAG<br>GAGCTGTACAGCGAGGAGGCCGTGAACGGCGAGACCCAGCAGGCCCACCTGCAAGCCA<br>TCAAGGAGCTGATCGCGCGCGACAAGAACCACCCCAGCGTGGTCATGTGGAGCATCGCG<br>AACGAGCCCGACACCCGCCCCCAGGGCGCCCGCGAGTACTTCGCCCCCCTGGCCGAGGC<br>CACCCGCAAGCTGGACCCCACCCGCCCCATCACCTGCGTGAACGTGATGTTCTGCGACG<br>CGCACACCGACACCATCAGCGACCTGTTCGACGTGCTGTGCCTGAACCGCTACTACGGC<br>TGGTACGTGCAGAGCGGCGACCTGGAGACCGCCGAGAAGGTGCTGGAGAAGGAGCTGC<br>TGGCGTGGCAGGAGAAGCTGCACCAGCCCATCATCATCACCGAGTACGGCGTGGACACC<br>CTGGCCGGCCTGCACAGCATGTACACCGACATGTGGAGCGAGGAGTACCAGTGCGCCTG<br>GCTGGACATGTACCACCGCGTGTTCGACCGCGTGAGCGCGGTGGTGGGCGAGCAGGTGT<br>GGAACTTCGCCGACTTCGCGACCAGCCAGGGCATCCTGCGCGTGGGCGGCAACAAGAA<br>GGGCATCTTCACCCGCGACCGCAAGCCCAAGAGCGCGGCCTTCCTGCTGCAAAAGCGCT<br>GGACCGGCATGAACTTCGGCGAGAAGCCCCAGCAGGGCGGCAAGCAGTAA
cadA **gene**: 2148bp	ATGAACGTTATTGCAATATTGAATCACATGGGGGTTTATTTTAAAGAAGAACCCATCCGTG<br>AACTTCATCGCGCGCTTGAACGTCTGAACTTCCAGATTGTTTACCCGAACGACCGTGACG<br>ACTTATTAAAACTGATCGAAAACAATGCGCGTCTGTGCGGCGTTATTTTTGACTGGGATAA<br>ATATAATCTCGAGCTGTGCGAAGAAATTAGCAAAATGAACGAGAACCTGCCGTTGTACGC<br>GTTCGCTAATACGTATTCCACTCTCGATGTAAGCCTGAATGACCTGCGTTTACAGATTAGC<br>TTCTTTGAATATGCGCTGGGTGCTGCTGAAGATATTGCTAATAAGATCAAGCAGACCACTG<br>ACGAATATATCAACACTATTCTGCCTCCGCTGACTAAAGCACTGTTTAAATATGTTCGTGA<br>AGGTAAATATACTTTCTGTACTCCTGGTCACATGGGCGGTACTGCATTCCAGAAAAGCCC<br>GGTAGGTAGCCTGTTCTATGATTTCTTTGGTCCGAATACCATGAAATCTGATATTTCCATTT<br>CAGTATCTGAACTGGGTTCTCTGCTGGATCACAGTGGTCCACACAAAGAAGCAGAACAG

	TATATCGCTCGCGTCTTTAACGCAGACCGCAGCTACATGGTGACCAACGGTACTTCCACT<br>GCGAACAAAATTGTTGGTATGTACTCTGCTCCAGCAGGCAGCACCATTCTGATTGACCGT<br>AACTGCCACAAATCGCTGACCCACCTGATGATGATGAGCGATGTTACGCCAATCTATTTCC<br>GCCCGACCCGTAACGCTTACGGTATTCTTGGTGGTATCCCACAGAGTGAATTCCAGCACG<br>CTACCATTGCTAAGCGCGTGAAAGAAACACCAAACGCAACCTGGCCGGTACATGCTGTA<br>ATTACCAACTCTACCTATGATGGTCTGCTGTACAACACCGACTTCATCAAGAAAACACTG<br>GATGTGAAATCCATCCACTTTGACTCCGCGTGGGTGCCTTACACCAACTTCTCACCGATTT<br>ACGAAGGTAAATGCGGTATGAGCGGTGGCCGTGTAGAAGGGAAAGTGATTTACGAAACC<br>CAGTCCACTCACAAACTGCTGGCGGCGTTCTCTCAGGCTTCCATGATCCACGTTAAAGGT<br>GACGTAAACGAAGAAACCTTTAACGAAGCCTACATGATGCACACCACCACTTCTCCGCA<br>CTACGGTATCGTGGCGTCCACTGAAACCGCTGCGGCGATGATGAAAGGCAATGCAGGTA<br>AGCGTCTGATCAACGGTTCTATTGAACGTGCGATCAAATTCCGTAAAGAGATCAAACGTC<br>TGAGAACGGAATCTGATGGCTGGTTCTTTGATGTATGGCAGCCGGATCATATCGATACGAC<br>TGAATGCTGGCCGCTGCGTTCTGACAGCACCTGGCACGGCTTCAAAAACATCGATAACG<br>AGCACATGTATCTTGACCCGATCAAAGTCACCCTGCTGACTCCGGGGATGGAAAAAGAC<br>GGCACCATGAGCGACTTTGGTATTCCGGCCAGCATCGTGGCGAAATACCTCGACGAACAT<br>GGCATCGTTGTTGAGAAAACCGGTCCGTATAACCTGCTGTTCCTGTTCAGCATCGGTATCG<br>ATAAGACCAAAGCACTGAGCCTGCTGCGTGCTCTGACTGACTTTAAACGTGCGTTCGAC<br>CTGAACCTGCGTGTGAAAAACATGCTGCCGTCTCTGTATCGTGAAGATCCTGAATTCTAT<br>GAAAACATGCGTATTCAGGAACTGGCTCAGAATATCCACAAACTGATTGTTCACCACAAT<br>CTGCCGGATCTGATGTATCGCGCATTTGAAGTGCTGCCGACGATGGTAATGACTCCGTATG<br>CTGCATTCCAGAAAGAGCTGCACGGTATGACCGAAGAAGTTTACCTCGACGAAATGGTA<br>GGTCGTATTAACGCCAATATGATCCTTCCGTACCCGCCGGGAGTTCCTCTGGTAATGCCGG<br>GTGAAATGATCACCGAAGAAAGCCGTCCGGTTCTGGAGTTCCTGCAGATGCTGTGTGAA<br>ATCGGCGCTCACTATCCGGGCTTTGAAACCGATATTCACGGTGCATACCGTCAGGCTGATG<br>GCCGCTATACCGTTAAGGTATTGAAAGAAGAAAGCAAAAAATAA
**PSAD promoter**:<br>822bp	GATCCCACACACCTGCCCGTCTGCCTGACAGGAAGTGAACGCATGTCGAGGGAGGCCTC<br>ACCAATCGTCACACGAGCCCTCGTCAGAAACACGTCTCCGCCACGCTCTCCCTCTCACG<br>GCCGACCCCGCAGCCCTTTTGCCCTTTCCTAGGCCACCGACAGGACCCAGGCGCTCTCA<br>GCATGCCTCAACAACCCGTACTCGTGCCAGCGGTGCCCTTGTGCTGGTGATCGCTTGGAA<br>GCGCATGCGAAGACGAAGGGGCGGAGCAGGCGGCCTGGCTGTTCGAAGGGCTCGCCGC<br>CAGTTCGGGTGCCTTTCTCCACGCGCGCCTCCACACCTACCGATGCGTGAAGGCAGGCA<br>AATGCTCATGTTTGCCCGAACTCGGAGTCCTTAAAAAGCCGCTTCTTGTCGTCGTTCCGA<br>GACATGTTAGCAGATCGCAGTGCCACCTTTCCTGACGCGCTCGGCCCCATATTCGGACGC<br>AATTGTCATTTGTAGCACAATTGGAGCAAATCTGGCGAGGCAGTAGGCTTTTAAGTTGCA<br>AGGCGAGAGAGCAAAGTGGGACGCGGCGTGATTATTGGTATTTACGCGACGGCCCGGCG<br>CGTTAGCGGCCCTTCCCCCAGGCCAGGGACGATTATGTATCAATATTGTTGCGTTCGGGCA<br>CTCGTGCGAGGGCTCCTGCGGGCTGGGGAGGGGGATCTGGGAATTGGAGGTACGACCGA<br>GATGGCTTGCTCGGGGGGAGGTTTCCTCGCCGAGCAAGCCAGGGTTAGGTGTTGCGCTC<br>TTGACTCGTTGTGCATTCTAGGACCCCACTGCTACTCACAACAAGCCAAA
**GDH2 promoter**:<br>1246bp<br>(The	TCGCCACCAGTCATAGACCATCAACAGTCATCGTCGACCTTTTCCACCAATTCGGGGCTG<br>TCGGAAATTCCTTACCCCGCATGTCGTGTCACGGCCGCGGGGGAAGCCTCCCCTGGGCTG<br>ATCCGCCCACTCCCTTTGAGCAATGGTTGGAGACCAAAAGCACGAGGTGTGGCGGCTGT<br>CGGTGTAGGTGACGTCCTCCCTGGCGGCTGATTAAGGGCCGATCAGATGCTGGCGAAGA

letters in **bold** represent GDH2-D2 sequence. The letters in red shading represent GDH2-D1 sequence)	GCTGGGCAAAGAGGGCGGTGGAATGGAGCTGCTCAGTCGCGGAGGCGGCGGCGCCGGC<br>AGCAGGACCGGCGCTGCAAAGCTGCTGGGCCTCCGTGTTCGGTGCACGCCGTCATGGAA<br>TACGGCACATACGTTCCAACTGCGGGCGGGGAGATGGGTGGGCGGGCTAGCTGACGGTA<br>AATCGGTGATTGGACCTGTGCAAGGTACTGTGTGCCGTGGGTGACTGCAGTGAGCCCCG<br>TGTCCTCTTCCCAGGCACTGCAAAATGAAGTGAGAAGTCGTGCCATGTGTGCATGTGATA<br>ATTGTGATTGCGTCCTTGTGTGTGGTGAGCATGGACAAGAAAGTAATTCAAGTATACGAC<br>AATCTATTGATTCTGCTGCTGGAGGGGAGGGTCACCGGGGCACCGTAGCATGCGGCGGC<br>GGTGGCAGTTGGCAGCTACGGCTAGCCATGCATGATGCATCCCCCCATGCACTGTGCGCG<br>CAGTTACGATGGATCGATGAGGCAAGGCTTAGGGCAGACCAGGCGCGGTCCCTTCATGG<br>TGCTTGTGCGATATGGTGGGACTGGTTTTCAACATGAGGCTGTGGTGAGACACCATGTCG<br>TCGGTACGCGTGGCGCGGAGCTGGTGCTGACGCGGGTGCTGGAAGTTGCGGGTAACGAA<br>CCCAACGACCTGGCGTGGTGCTGGCTCATTCTGGGGGCGCTGG**TCTCGGAGACCACTC**<br>**CAATATTGTCAAAATCCTGTTTAGACAATGTTGCCGTGCTGCCACAGGCCGGGCAG**<br>**CTAGAGGAAGTGTCAAGCGTGCATGGGCGCTGACACGTTTCCGAGCTGGATGGTC**<br>**GTCGCTGGACTCTCAAAAGTCCACGAACCTAGCGCCCGCAGCCATGTTGCACATTC**<br>**GTCCGGTTGAGCTCGTATAACACATCTATTCACTCGTTTTCAACATACGTCTGGCGT**<br>**TACCGAACCTCTTTCGCCTGCGAGCTGCGTAAACAAGAGGCCAGCCCTGGTCGGG**<br>**GGAGTTTGCGCAGGCTGGC**
**LHCBM3 promoter**: 1542bp	CACTGCACCGAAAGGTGTAATGGATGGAAGGACCAGGTAGTTGTGAGCAGTGAGCGACG<br>GGTTATGGCTGTAGTGTGGTGGGAGGCGCGTGGGCCGTGGACACCAGACGCGAGGGCTG<br>TGGGGCTCTGACGCACACGGAGGAGGAGGCACCAAGGCACGGCGGCCTGGGCCCAGGG<br>CGCGATGCCACACGGTACTGAGAGTGCACTGACCAGGCGGGCGGGGAGGAGGGGCGGG<br>CAGGCACCTACGCCCCCTGCTGCCCTTGTAACCCGACCGCCCGACCAGCACCACTGCAC<br>TGCCCGTGGCAAGGCCTGCTGTGGCACTCATGTGCGCACTGCGTTCCCCCCCCCCCACA<br>CACACACACAGCCACGCACAGCCAGCCAGCGGGCTGCGGCGATACCTGGAACCGTGGC<br>AACTGGCAGTCGACATCTGTTGGCACCGCACCGCACGCCTGCGTCGTCTCACGCACTGG<br>CGCACCGGCTCGGCGGCGAGGGCGGCGCCTGCCAGCGGCCGACCAAGTCCCAACCACC<br>GCACGTCGGTCGCCAACCGCTGCAAATCAGAGCCGACGATGCGCGCTGACCCCGTGTCA<br>TGTACGGGGCTCACGGCTGTTCTTAAGCGTGCGGGTCGCGAGGCTCGCGGCGTGTCGGG<br>CGGCCCCTTGTCAGCGCTGCAACGCTCGGGCTCTGGAGCGGGCCGGCCCGCAAGTCGAC<br>AGGCCGTGGTGCCGGCTGGCCCTAGGGCCGCGCCCTGATGCGAGCGCTCAGTTGCCGAC<br>TAGAAAACCCTTGCTGCGCTTCCCTACACAACAACAGCGCCGACCACATCCTCGCTTGCT<br>GTGCCACGCACCGAACTCGCTTCAAACCCGTGTCGGCACTTCTGAACACGCGCATCGCC<br>GGACCGCCGGTTGCTGACGCCACCCCCAGCCTTTAGGTAAAGTATTCCACCACATCCTGC<br>TGATGCGCTTGCTCGCTAGTCCATCAGACTTCGATATCATCCAGCGGTGGCGCGAAGCAG<br>GAGCGCCCGTCGACATGCGCGACTGCACTCGCACCAAGCTAGCGGCTGAGCCCAAGCCC<br>AACATCTAATTGGCGCTGTTGCTGGGTATGAAGTTAGCGCTACACTCAGCCCGATGAAGA<br>GTTTATGACGAGCGACGGTTCAGATGCGTTCCGGAGCTGGACCACCGTCACGCAGAGCC<br>CCAACAAAGGCTCCACATGTGCTAATGCAAACAACATGTATCACTTGTAACCAGCAGCTT<br>TGCCAATGTGCATGATTTGGCTTAGCGAGGCAGTCGCAAGCCTGGCGCGTGGCGCATTCT<br>TGCAGCGCGCCAGCCCCCGCAGCTTTAGCGGTCCATTGCAAGCTCAATGGGCCACGTCG<br>CAATGCACCAGCGGGCGAGCGGACAGCGCACGCTGGCGCGCGACCGATCGGGCGCGGG<br>GTCGATCGAGATGGCCGGGTACGGCGATCGGCGCGCGAGATGGGACCATGAACCCCTTC<br>TTAAGCGACGCGCGCTCGTGAAAATATCGTGACCGGCTTCAACCAGTCCCCACACATCAG

	TCAAA
**AR promoter**: 461 bp	TCGCTGAGGCTTGACATGATTGGTGCGTATGTTTGTATGAAGCTACAGGACTGATTTGGCG<br>GGCTATGAGGGCGCGGGAAGCTCTGGAAGGGCCGCGATGGGGCGCGCGGCGTCCAGAA<br>GGCGCCATACGGCCCGCTGGCGGCACCCATCCGGTATAAAAGCCCGCGACCCCGAACGG<br>TGACCTCCACTTTCAGCGACAAACGAGCACTTATACATACGCGACTATTCTGCCGCTATAC<br>ATAACCACTCAGCTAGCTTAAGATCCCATCAAGCTTGCATGCCGGGCGCGCCAGAAGGAG<br>CGCAGCCAAACCAGGATGATGTTTGATGGGGTATTTGAGCACTTGCAACCCTTATCCGGA<br>AGCCCCCTGGCCCACAAAGGCTAGGCGCCAATGCAAGCAGTTCGCATGCAGCCCCTGGA<br>GCGGTGCCCTCCTGATAAACCGGCCAGGGGGCCTATGTTCTTT
**ACP2 promoter:** 1460 bp (The letters with underline represent ACP2-D3 sequence. The letters in **bold** represent ACP2-D2 sequence. The letters in red shading represent ACP2-D1 sequence)	GGGTGCAAGCCTGGCCTGCACGCCCGCCTTCACATGCACGCTCGGTGGTGGGGGCGTAC<br>AGGAGGCGGGCCTGGCCAGGGCTTGCATCTTCCGGGAGCGGTGCTTCAGCCCCTCGGGC<br>GAGGATGTTTGGGCAAGGCCGGGGTGCATTCTCGCCGCGTGGGGCTGCATACGGTCCCC<br>GAATCTCGCCAAATCCCCCTTCCCGCGAGCGGGTTTTGGGGGGAATCCTAGTTTTTGGCT<br>CCGCCCCCCCCCCCAAACGCTGAAAACTGGGCACTTTTGGCCCCTGCATGCCGTGGGCG<br>AGAACGTGGTCAGGTCCCTTCCCGCCTGCGGCGGCGGCCCACCCCTAGCCCTGGGCCTG<br>GCCCGAATCCTCATATCGTGCCTCATGTCCCCGCACTCCCTCAGTCCACACCACTTGCCCA<br>CTGCTGGGTATCCCTATTTCTAGTTTTATTTCATTTCATCGACGAGGGCAGGCCGCTTCGG<br>CGGCCGATAGCCCACATACACCCCGCTAACCGACACCCAGTGTCGTCACGCATGAGGCG<br>GAGCGGTCCCGTCACTGCAGTGGAGAAGCCCCCCACCCTGGATCGCTGGCTCAGCCCAA<br>AGGACCGCAGACATATGCAGTCAAACCTGTGTGCTTAGCCTAAGCGCCGCTTCAGTAAG<br>GCTCGCTGTAACGGCTGAACTACACAACGCTTAGGCGCTGCTGGCGGGGGACATGCGCA<br>TGGACATGTCTGGACCGGAGCACGTCCAGCAGGGCCCCTTCACACTGCAAATAAATGAC<br>ACAAATGACAGATAGGATGCTTGAAAGCGACGCGGCAGGTGCATTGTGGATGGCGGAAC<br>CAAGTGTGCTATGCTTCTGAGGGCCTCGACTAAGCGTCGCTTCGATAATGCTATCTAAAAC<br>AGACAAACAGCATAGGACCTGAGTTCGGGTCATGAAAGCGCTTGTCTGCTAACAGGCGT<br>CGGAACTTGCTGGGGTTCTAGAAAGGTAGAGGTGTT**GCCCTACAGATCGGCTAACTGT**<br>**TTAAGAAACTATCGTTCCAATAACGGTGCAGAAGCTAACAGCCAATTCGGCAAGCG**<br>**CGTGTCCTTGTGACTGGGCGCTTACGACTGTTTTCAACGCTTCAGCGCGCCTATGA**<br>**CTTCTCCATGTTTCTAAACAACAACATTTCCTTGTGGTATTTGCGTTGCAGTGCATG**<br>**GTGCTCGGAGCCTAAGCGCGAACTCGCAGGCCGTGCAGGCGTTGGGGCTTTGGTG**<br>**CAAGGCATTTTGGGTCAGCTTACTACTACATGCAAACGCTTGCACCATGAGCGTAAT**<br>**TGGCAATGCATCAGGGCCCACCGGTCGTTCTGCGGGCGTTTGTACCGCGCATGGGT**<br>**CGGCGTTGGCATGGGCAGCGGGCGAGCAGATAAGCGCCCCGCGCCCGGACGTGCA**<br>**ACCTACACTTCCCACAGCACCCAATCCTTCTCTGCTACTTTGCAGCCCAATCCACTA**<br>**CA**
**PETM promoter**: 1088 bp	TCAGGCGATTGCAACACCCTTGCGACAGCTGCAGCAAGCCCCTCGGTGACAAGCCAAAC<br>GTGATGCCTATAGTCTGCTCATAACAATTGAGGACCAGGGCTGAAGAGGTGCTGAAGTCG<br>AGTTAAAGTTCGATATTCGCGTCCATGAACGCGATTACTGGGGACAGTGTACGCTAGCAC<br>CTGTAACCTGTAAGTCCTAATACTTGCTCTCTCGTATGCGCAAATCCGCCAAAGCAGGCG<br>AGCAGCGGCACACCATGCGGTCCTCACCCTCTCCCGCACACGCTTGGGCCCCCGCCCCC<br>TCCCTTGCCCGGACCCTGGTCTCTGCCGCCCATTCCGAGAGCTAATCTTCTGATTTCGACG<br>CCGAATGATAGCATAGCTGCAAATCCATACTAAAGGAGTTAGCAGGGGCGAAACAGGCT<br>GACTGAGATGGACTGTGCCTGGATGCCACCACATGCTGCTGTGCGTGCGACCTTAGGAGT

	ACGCTTTGCCCCCCTCTGTTCTCATCTATATCTCTTGGCGCGAAGCGCAGTGAAGCGCTG<br>ACAAGGTAATGAGGTCTCCTGCTGCGCACAATTTACCCCAAGGTGGATCCTTTGGCGCTA<br>CAGAAAAGGCTGTGGTGGACATTGGGCGGGGTGCGAGAGTTTGGGTAGAGCGTGGCAG<br>GGAGGGAGGACTTCAAGGGGAGAGGAAAGTGACGCCGGTAGGCGGTAGGCCCTAGCCG<br>GTCCCACGGTTCGAGGGCGCGCGTCTCGCCCGCTTTCCCGAAGCAGAGGGGTATGGAGT<br>CAGAGGCGCTACAAGTGAGGCGTTTACATAAGTTTTAGGCGGCAAGACGCGGCTAGCGG<br>CGACGCAGTTGGCCCGCAAGCCTCCAACGGGAAGCGAAATAGCGCAGGACACATTTTTT<br>TACAACAATGTAAGGTGCAGTATGTACGCATATCAGTCCTGTATACCAAGGTATTTATTACA<br>GGCCCCAATTCGGTTGCGCCAATCGGTACGGTGAGGATGGGCCCTCGCCCCGCAAGGAT<br>AGAGCCCGCGGGCTTGACTGTCTCACCACGCTTCCAGTAGTCCACCGAGCCCGTACTTCT<br>TCTATACAAGACCAAC
**FAP12 promoter**: 2143bp	TGCGTTGCGTGGTGACACATACGGTAGCATTCGGTATACGGTACATACATGCATCTTGACC<br>GCACCCGCTTCTGGAACTCCCTAACACCAAGGTGACCACTCCCCGGTCACACAGTCGTG<br>GCGGTCCTCTCCTCCGCCATGCCGCTTCTCCCACCCTGGGAGGTTCACCACAGCCACAGT<br>CTCCCCGGCCCCTACATCCACCTGCCACCCACCAGCCCGCTTCCCCCCGTCCTCGTGTCT<br>GTTATTGGCAAAGATGGACACAAGCCCTTCTTCCCGCACGCACCTGGGTTGACCACCTCC<br>TCCGGCTCGAGCGGCCGCCACGCCAGCAGGTATGTAAAGTCCTCTAGCGGGCTGTTGCC<br>CTCCGTGCCGCCGAAGGTGTACGCCACACCCGGCACGCAGTGCTGCTTGCGCTCCCACA<br>CGTGCATGCGCGCGTTCTTGATCAGGGCCAGCTTCTGACTTAAGATGGCCAGAACCTGGC<br>AGATGGACGAGAAGGGAAGCGTTGCGTGCGTGTGCAGTGGTGGTTCGCCGGGGGTTAC<br>ACCGCCGCCATAAGGCGCCGCACATGGACACACATACACATACAGCGCGTTTCTATAACT<br>ATTAACACAACATACACGCCATACAAACACACGACCCGCCGCCGGCCCGATTGCGCTGCC<br>ACCCACTCACCTTGGTGTGCAGCCCAAACTGCCAGTCCACGGGCATGGACGCCTTGCTC<br>TCCGGCAGGCGGTTGAAGTCATTTCGCAGCCACTGGGGCACCTTGGTGGTGCTGGTCAG<br>CAGCACGTAAACCTGCCGGGGTGAGGACATGAGGAGATGTAAGCAGATGTTGATAGGGG<br>TTTGAGGCAGGAGCAGGAGCAGGAGCCCTCCCGCTGGCCTCTGAGCCACCCACCGCACC<br>TGCACCGGCTGAAAGAAGCTGGCGTGCATGTAGTCCGGCCGGGCGAACGTGAAGTGCTC<br>ATTGACGGCTGTGAAGCAGTACACGCACTTGTCACGAAACACCTCGGGCATCTCGGCAT<br>CGGACACGGCGGCCAGCTTGTGGGAGGTGGTGTCCCACAGCGGCCGAGAAGCGTACTC<br>CTTGCAGAAATCAAACATGGACGCTGGGTCGACGGGCACACCCTGGGAGTCGCGGCCAT<br>CGGAGTGCAGTGTGAGCTCCAGGATTCCGCGCTCCTTCATGCCAGGGGCGTGGCCGGCC<br>GGCGTCGGGGGCACCTCGAGGATGGCGGGGCCGCCCGTGGAGATGCGCACGTTCCTCCG<br>TGGAGCACCCTCCTCCGCGGGCTCCGACTTGCCCGCAGGCATTTCTGACACTTCGTCAGA<br>AGTTTTCATGTTTATTACGTGGGTGTCACCACGGGGCCCAGGGCCCTTTTGCTCCGAGGA<br>ATTCAAGTCCCAAGCGAGTTTTCGAGACGCGCCCCCAAGCGCTGCTGTCGACTCAGCTA<br>ATGCCTTGCGTTTGTCTCCTATCTGCTGCAGTTGGCACAGCGCGCAACTGAACACGACTA<br>GCTAGCGCGTTCGTAGAGTCCTAAGCGTGCGAGCATGAAGGGGGAGCTCTTTTATTGATT<br>AGTCTCCAGCTGTTGCAAGAATGCGAAGACAGCTACACGTAGTTCGTCCACTTTGACAA<br>GTAGAGTTTCTGCTTGCAGACATTCATCGCGGTTACAATATCACAATATTGCTGTCGCCCG<br>GGTAAATGGAATGCTCAATGGCTACAGCTGAAAGCATTGTCTTATTCGAAGTGACAGAAG<br>CAGAAAACGTAGAAGCCCGCAAAGGGGGGGCCCGCCGGGTCGCAACGCGCCATGATCG<br>GGGCAGTTAAGTCGATCGGGGCAATCCGCGCACAACCTAGCCCAATCAATATAGCTTAGC<br>ACATTACATAAGCAGCAGGGCTGGGTGGCTGCGCTGGCGTTGTCGGGCGGGAACAAAGT

	TGCTCTAAGGCCGACATCGAAGGTTCTGCGGATTGTTCGATGGGCCCCACATAAACGACT<br>GCCTGCTTGCAGTTGATACATAACAGCTGTGCTGCGTTCATAGACAATTGTCACTGACAA<br>GTCTCAGTATTAGAACGTGATACTCAAGCACACTACTCGCGCACACCATCTTGCTTCCTCG<br>AACAGTCTTTCCAGGAAAACAACCTTGGCACAGAGTCGCCACGAGTTAAGGCGTATCAA<br>CC
**FAP310 promoter**: 1083bp	TGGTCCGTGTGCAAGCACGCCGGCTGCAACTCCAAGTCGTCCCAGGGCGTGCCGCTGCT<br>GGCGGAGCAGGCTTCCAGCTTCTTCTCGTCCAAGGCCCAGGACGCCTGGCCCGCCTCGC<br>TGGAGGATGCCAAGACCATCAGCTTCAACACCTGGTGCCGCCACGAGGACGTTGAGGCC<br>TTCACCATTCCTCTGTACACCGCAGCTGGTGACGAGATGAAGGTCGCCGCTCAGGTGGGT<br>GCGCACTGGCGTGTAGAACACGTGCTTGTGGGTGGACGCAACGTGACTCGCACACATGA<br>AACACCGTAACAGCACCCCACAGCGTGTCCACAACATTTATCCTTGCTGTCGTGCGTTCG<br>CTCATGTTTCGCTCTCTTTGCTCCCGCTCCCTCATTCCTCAGGCATTCGGCAAGGCTGGCG<br>TGTCCGGCGCTGTGGTCGTTCTGGGCTACAGCTGGAAGGAGGGCGCCAAGGCCCGCTGG<br>GGCGAGCTGCTGGAGAAGCTGGTGTCTGACTTCGCCGCCGGTGCCTACGTGGTCCCCGC<br>TCAGGGCAACGCTGACGAGCACCCCGAGGCCGTTGACTCCGTTCTGGAGTCCGCCGCTG<br>ACGTGGCCGATGAGGCTGCCGAGGTGGTGCGCCGCTTCCTGCAGAGCGCCGCCGGCACC<br>TACCCCTCCCCCGCCCCCACTCCTTCCCCCTCGTCCTCCCCTTCCCCCTCTCCCTCCCCCT<br>CCTCCTCTCCCTCCCCCTCTCCTTCTCCCTCTCCCTCCCCGTCTGCCTCCCCGTCGCCCTC<br>GCCCAGCCCCTCGCCGACGGCCATCCCTTCGCCCAGCCCGCCCTCGCCCCTTCCCCCTAG<br>CCCTCCCCCCCTGCCCCCTGGCATTGCCAAGGTGGTCGAGGACTTCGTGGTCACTTATCA<br>GAACCAGACCATCGGCGACCAGGACCCCGCGGACCTGCAGAAGAACCAGACCGCCATC<br>AACAGTCTGGTCGATGACATCCAGAACACCTACGCGAAGGCCCTGGGCGTCAACGCGAC<br>GGCCATTGTTGTGCGTGGCTTCCTGTTTGTGTACCGCAACGGCACCGTGATCACCATCAC<br>GGTCGACACCA

Supplementary Table S2. Information on the plasmids constructed in this study.

Plasmid	Backbone	Promoter	Gene to express
1	*pJ1DCF*	AR	*GUS*
2	*pJ1DCF*	PSAD	*GUS*
3	*pJ1DCF*	PETM	*GUS*
4	*pJ1DCF*	GDH2	*GUS*
5	*pJ1DCF*	LHCBM3	*GUS*
6	*pJ1DCF*	ACP2	*GUS*
7	*pJ1DCF*	FAP12	*GUS*
8	*pJ1DCF*	FAP310	*GUS*
9	*pJ1DCF*	ACP2-D1	*GUS*
10	*pJ1DCF*	ACP2-D2	*GUS*
11	*pJ1DCF*	ACP2-D3	*GUS*
12	*pJ1DCF*	GDH2-D1	*GUS*
13	*pJ1DCF*	GDH2-D2	*GUS*
14	*pJ1DCF*	GDH2-D1/ACP2-D1	*GUS*
15	*pJ1DCF*	GDH2-D2/ACP2-D1	*GUS*
16	*pJ1DCF*	HSP70A/ACP2-D1	*GUS*
17	*pJ1DCF*	PSAD/ACP2-D1	*GUS*
18	*pJ1DCF*	GDH2-D1/PSAD	*GUS*
19	*pJ1DCF*	GDH20D1/ACP2-D1	*GUS*
20	*pJ1DCF*	GDH2-D1/ACP2-D2	*GUS*
21	*pJ1DCF*	AR	*cadA*
22	*pJ1DCF*	GDH2-D1/ACP2-D1	*cadA*

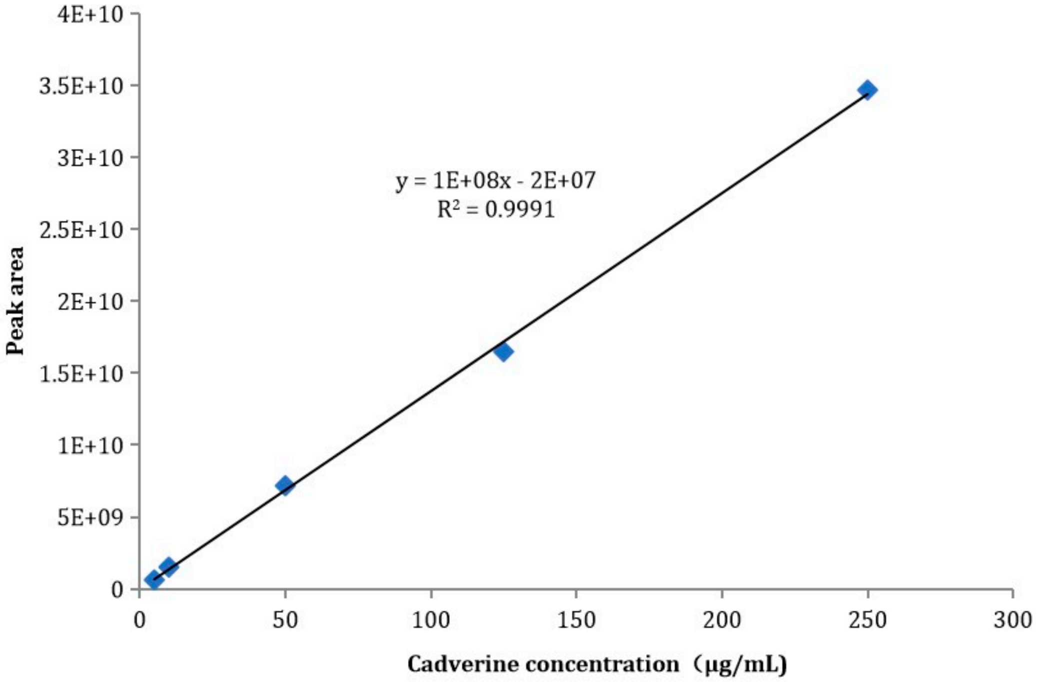

Supplementary Figure S1. Standard curve of cadverine concentration against peak area.

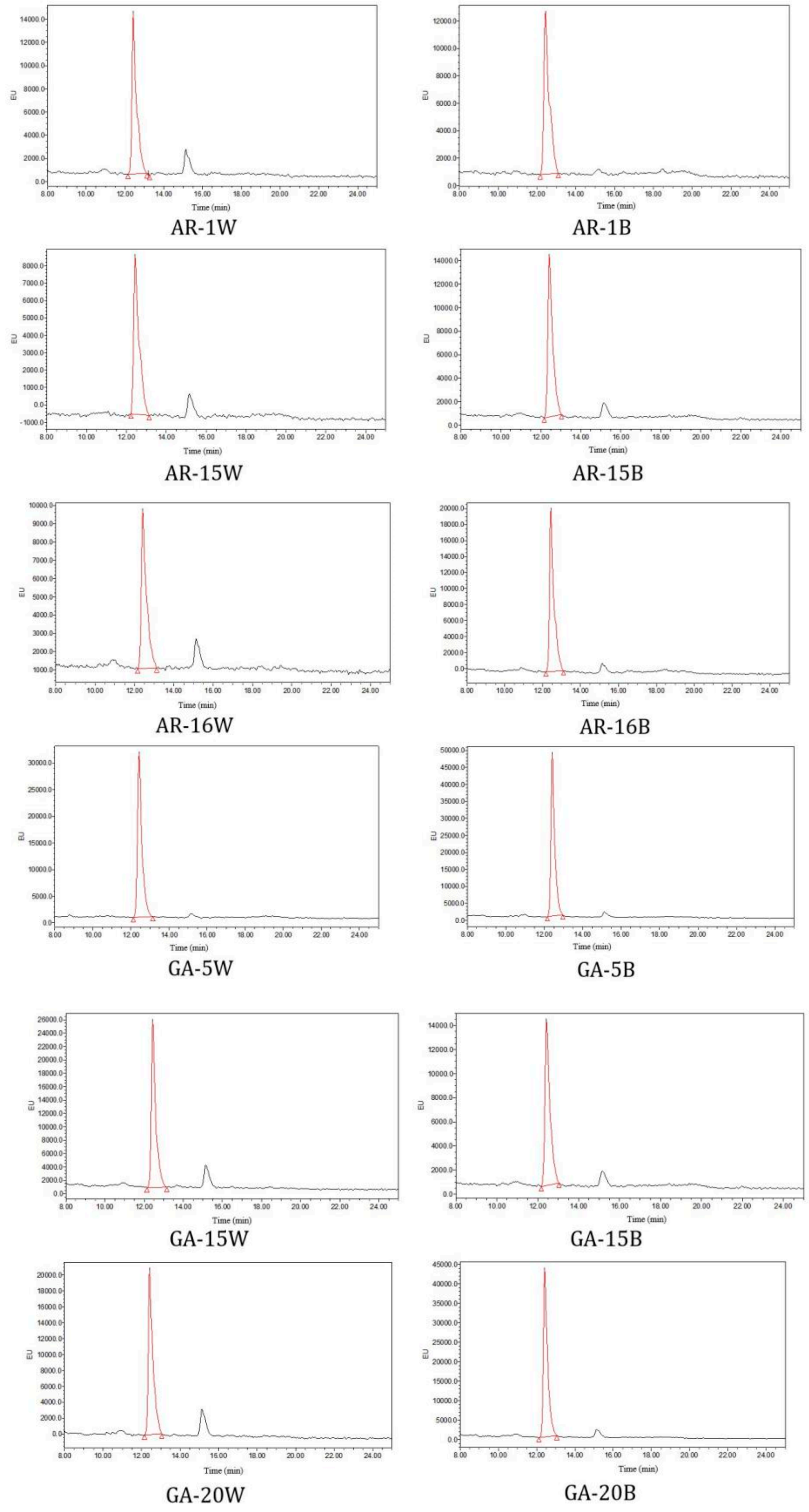

Supplementary Figure S2. HPLC spectra of cadverine measurements of different experimental samples. The peak of cadverine showed up at 12.5 min. AR, experimental groups with AR promoter. GA, experimental groups with GA chimeric promoter. W, white light. B, blue light. The number after the dashed line represents clone number.
